# The impact of internet use on physical activity among Chinese older adults: the mediating role of social support

**DOI:** 10.3389/fpubh.2025.1492188

**Published:** 2025-01-23

**Authors:** Yifei Shen, Chuntian Lu, Bin Wang

**Affiliations:** ^1^Department of Physical Education, Xidian University, Xi’an, China; ^2^Department of Sociology, School of Humanities and Social Sciences, Xi’an Jiaotong University, Xi’an, China; ^3^Department of Management, Wuxi Institute of Technology, Wuxi, China

**Keywords:** internet use, physical exercise, social support, older adults, mediation analysis

## Abstract

**Background:**

The positive impact of Internet use on enhancing physical activity among older adults is an important response to the aging problem in the new era. However, while previous studies have explored the important impact of Internet use on physical activity among older adults, the specific mechanisms of this impact have not been much analyzed. In an attempt to answer this question, we further focus on the mediating role of social support in the relationship between Internet use and physical activity among older adults.

**Methods:**

This study utilizes data from the 2021 China General Social Survey (CGSS) to categorize social support into three dimensions: support from relatives, friends, and neighbors. A nested multiple OLS regression model is employed to analyze the impact of internet use on physical exercise among the old adults. Additionally, the SPSS macro PROCESS is used to test the mediation effect, examining the mediating role of social support in this relationship.

**Result:**

Our research findings indicate the following: (1) Internet use significantly and positively predicts physical exercise among the old adults; (2) Social support exerts a positive influence on physical exercise in this population; and (3) Social support partially mediates the relationship between internet use and physical exercise among the old adults.

**Conclusion:**

The study concludes that increasing Internet penetration among the old adults is of great practical significance in expanding their social support network and promoting physical activity. The results of the study provide new perspectives on the layout of work on ageing and policies for healthy ageing in the new era, as well as lessons for other developing countries on the issue of ageing.

## Introduction

According to data released by the United Nations, the global ageing trend will continue to deepen, with the proportion of the world’s population aged 65 and over more than doubling by 2050 (1). Unfortunately, China’s aging problem faces greater challenges and has become an increasingly prominent issue in Chinese society (2). According to statistics, as of 2020, China’s population aged 60 years and above had reached 264 million, accounting for 18.7% of the total population (3), and it is expected that by the end of 2025, this number will rise up to more than 300 million (4). The aggravation of population ageing goes hand in hand with the gradual decline of the physical functions of the old adults, resulting in a huge challenge to the health status of Chinese national old adults population (5). In order to cope with the health problems well that by population aging, in the Opinions of the Central Committee of the Communist Party of China and the State Council on Strengthening the Work on Aging in the New Era, released by the Chinese government in November 2021, sport is listed as one of the important initiatives to cope with the problem of aging, and older people are encouraged to be active in physical exercise to improve their health status. A large number of previous studies have also confirmed the role of physical activity in preventing diseases (6), enhancing the physical health (7), mental health (8, 9), and physical fitness (10–12). Therefore, how to promote physical activity among the old adults to help the development of a healthy China and healthy aging is a social issue that scholars should pay attention to.

With the continuous advancement of technology, the advent of the internet era has significantly expanded opportunities for individuals to access information, breaking traditional spatial and temporal boundaries and profoundly transforming the lifestyles of older adults (13). In China, internet usage among the old adults has become increasingly prevalent. According to the 54th Statistical Report on China’s Internet Development, as of June 2024, the total number of internet users in China reached nearly 1.1 billion, an increase of 7.42 million compared to December 2023, with individuals aged 60 and above accounting for 20% of the new users (14). Relevant studies have shown that older adults use the internet not only to strengthen interactions with friends (15) and enhance their subjective well-being (16) but also to access information related to physical exercise (17) and health (18). Such information provides knowledge and skill support for their participation in physical activities, boosts their motivation, and ultimately improves their physical and mental health (19–21). Furthermore, the State Council of China, in the National Fitness Plan (2021–2025), explicitly advocates for the deep integration of the internet with physical exercise, promoting the “Internet + Exercise” model to increase public participation in physical activities (22). Therefore, with the intensifying trend of population aging, the internet has emerged as a new factor influencing the participation of older adults in physical activities.

Researchers have recently begun to focus on the relationship between internet use and physical exercise among older adults. It has been noted that internet use can help older adults establish new social networks (23), thereby promoting their participation in physical exercise. Similarly, Sasaki’s research found that the effect of internet use on encouraging physical exercise is more pronounced among older adults with limited kinship interactions or lower levels of social participation (24). However, through what pathways does internet use influence the physical exercise behaviors of older adults, and what are the specific mechanisms at play? These questions remain to be further explored. In this context, social support may serve as a critical influencing factor (25, 26). On one hand, previous studies have emphasized the positive correlation between social support and the quality of life of older adults (27–30). On the other hand, the internet has been shown to impact social support (31–33). Therefore, internet use may not only have a direct impact on the physical exercise of older adults but also exert an indirect effect through social support. Based on this premise, the present study focuses on the impact of internet use on physical exercise among older adults, with particular attention to the mediating role of social support. Our findings aim to provide new pathways for promoting physical activity among older adults and offer actionable policy recommendations and implementation strategies to address the challenges of healthy aging.

## Literature review and research hypotheses

### Internet use and physical activity

The internet has now become an integral part of everyday life, and evolved into a lifestyle (34). Internet use among the old adults refers to the engagement of individuals aged 60 and above with the internet via devices such as computers (33). Scholars have noted that the internet serves as a primary source of health information (35), and also provides a communication platform for vulnerable groups (36). For older persons who are a vulnerable group, the use of Internet platforms provides access to a wealth of factual information, instant interaction and a variety of convenient services without having to leave their homes. It helps them to gain more health knowledge and social support. Research from various countries has also confirmed that internet use contributes to improvements in the physical health, mental well-being (37, 38), and life satisfaction of older adults. Nowadays, internet technologies are increasingly applied in the realm of physical activity, expanding the channels for disseminating information on sports and exercise. Therefore, older adults are gradually using the internet to access information on health and physical exercise, thereby improving their healthy lifestyle (39). For instance, Kettunen, Kari, and Frank suggests that older adults can engage in physical exercise through sports apps (40). Additionally, Guo’s research indicates that activities such as “reading news,” “chatting,” and “watching videos” significantly increase the likelihood and frequency of old adults individuals participating in physical exercise (41). Wang et al., using data from the 2017 China General Social Survey, found that internet use has a significant positive impact on increasing physical exercise (42). In summary, although the direct influence of internet use on old adults participation in physical exercise is well-established, the underlying mediating mechanisms remain unclear.

### Social support and physical activity

The concept of social support was formally introduced as an academic term in the early 1970s and has since been widely used to describe the various forms of assistance and aid provided by family members, friends, neighbors, and others (43). Bernard conceptualized social support as a collective network of family, friends, and social institutions that individuals rely on to meet their social, physical, and psychological needs. Functionally, social support encompasses emotional support, instrumental support, informational support, and companionship (44), and represents the spiritual and material assistance individuals receive from their social networks. From a structural perspective, social support can be categorized into formal and informal social support (45). Formal social support is provided by governments, institutions, communities, and other official organizations, such as pension schemes and healthcare security systems (46). In contrast, informal social support refers to networks based on geographic and kinship ties, including material assistance, emotional support, and informational support from family members, friends, relatives, and neighbors (47, 48). Research by House and Turner has demonstrated that meaningful groups surrounding individuals—such as family members, friends, colleagues, relatives, and neighbors—offer significant support and positively influence individuals by providing practical help, social–emotional assistance, and informational support (49, 50).

Scholars have explored the impact of social support on physical exercise from various perspectives. Previous studies have identified that social support exerts a positive influence on old adults participation in physical exercise. For example, Iso-Ahola and Park point out that emotional support within social support can promote older adults’ participation in physical activities and help them maintain regular exercise behavior over the long term (51). The findings of Loprinzi and Joyner also support this view, further revealing that the presence of emotional support increases the likelihood of older adults engaging in physical exercise by 41% (52). Furthermore, scholars have found that establishing a strong social environment and subjective emotional support system can effectively motivate empty nesters to participate in physical activity (53). Therefore, although scholars have recognized the influence of social support on physical exercise, the impact of different dimensions of informal social support on promoting physical activity among the old adults has received less attention. Therefore, given data availability and research objectives, this paper focuses on “informal social support,” defined as the potential, mobilizable relationship resources with social interaction functions in the daily lives of older adults, including support from relatives, friends, and neighbors (54).

### Internet use, social support and physical activity

In recent years, with the widespread use of the Internet, scholars have begun to focus on the relationship between Internet use, social support and physical activity. On one hand, regarding the research on the relationship between Internet use and social support, some views believe that the Internet, as a powerful communication tool, opens up a new social space for socializing to help people maintain established social relationships and can develop new ones (55), and thus improves the public’s social support, social identity, and sense of belonging (56). For instance, Quan’s research indicates that the internet can assist older adults in seeking social support (57). Guo’s study found a significant correlation between specific types of internet use—including online chatting, gaming, and entertainment—and social support (41). Heo et al. found that older adults who successfully create and share content online enhance their social connections, particularly with relatives and friends (31). A study from Finland also highlighted that the internet serves as a source of social support, aiding individuals in obtaining emotional and informational support (58). On the other hand, communication studies suggest that online media expand the modes of physical activity participation, enhance interactions and relationships within sports-related networks, and enrich the content of sports communication, thereby improving residents’ engagement in physical exercise (59). The “networked society theory” posits that internet use has a positive impact on older adults by broadening their social networks and promoting social participation (32, 57), thus providing more opportunities for physical exercise. It suggests that internet use can further expand older adults’ interpersonal and social support networks, potentially increasing their motivation for physical activity through this mediating domain. However, previous research has generally examined these factors in isolation, without integrating internet use, social support, and physical exercise into a unified framework. Therefore, this study aims to explore how different dimensions of social support influence the relationship between internet use and physical exercise among the old adults.

### Research purposes and hypotheses

**Figure 1:**
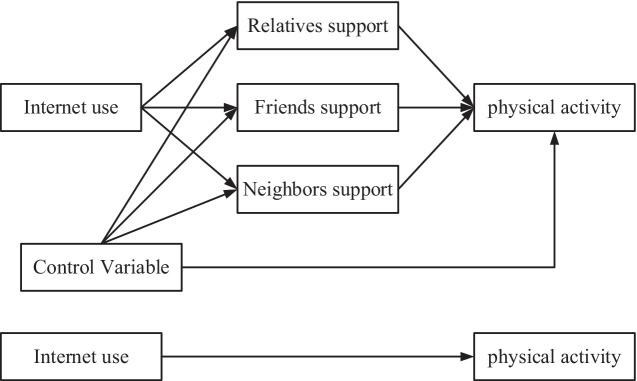
The hypothesized model.

This study aimed to explore the relationship between Internet use, social support and physical activity among Chinese older adults, and focused on whether different dimensions of social support mediated the relationship between Internet use and physical activity. Therefore, this study was divided into the following three steps. First, the nationally representative CGSS survey data were used to analyze the impact of Internet use on physical activity among older adults through multiple linear regression models; second, the impact of three types of social support, namely, relatives, friends, and neighbors, on physical activity among older adults was analyzed through multiple linear regression models; and lastly, multiple mediation analyses were conducted to explore the relationship between Internet use and physical activity among older adults through the three dimensions of relatives, friends, and neighbors’ social support indicators to influence the path of physical activity of the old adults; based on this, we propose the following hypotheses (Figure 1):

H1: Internet use has a positive impact on older adults’ sport participation.

H2: Social support has a positive effect on older adults’ sport participation.

H3: Social support has a mediating effect between Internet use and older adults’ sport participation.

## Methods

### Data sources

In this study, the above proposed propositions are validated using the Chinese General Social Survey (CGSS) 2021 data. The Chinese General Social Survey (CGSS), conducted by Renmin University of China in collaboration with academic institutions around the world, is China’s first large-scale nationwide and comprehensive social survey program, which has been widely used in the field of social sciences. 8,148 samples were completed in the CGSS 2021, and the CGSS 2021 survey contains data related to residents’ Internet use, which is a rare and representative data on the use of the Internet by individuals in China. The samples selected in this paper are old adults people aged 60 and above, and 2,885 valid samples are obtained after removing missing values and invalid samples.

### Measures

#### Dependent variable

The dependent variable of this study is the participation of older adults in physical activity. According to American scholar Kenyan’s definition of the concept of sports participation, sports participation should include four levels, namely “cognitive,” “affective disposition,” “direct participation” and “indirect participation” (60). In this study, residents’ sports participation was limited to the “direct participation” level, i.e., direct participation in sports activities. In the questionnaire, respondents were asked “In the past year, did you regularly participate in sports activities during your free time? The options were, in order: 1 “every day,”2 “several times a week,”3 “several times a month,”4 “several times a year or less,”5 “never.” For the purposes of this analysis, we combined and reverse-assigned the answers that “never” or “do not know” (=1), “a few times a year or less” (=2), “a few times a month” (=3), “a few times a week” (=4), “every day” (=5). Larger values indicate more frequent participation in sports.

#### Independent variable

The independent variable is Internet use. According to a study by Han et al. in 2021 (58), We chose the question “In the past year, what was your use of the internet (including mobile internet)?” to measure the use of the Internet in CGSS. Based on the respondents’ answers, the rating in terms of the frequency on a 5-point scale ranged from “never” (=1), “rarely” (=2), “sometimes” (=3), “often” (=4), and “very frequently” (=5). Higher scores indicate more frequent Internet use among older adults.

#### Mediating variables

The mediating variable in this paper is social support, specifically informal social support. We use Berkman and Cantor’s conceptual framework (61), which describes informal social support in terms of three key dimensions: kin support, friend support, and neighbor support. In the CGSS, we used questions in our questionnaire such as, “In the past year, have you often met with relatives you do not live with in your spare time?” “How often do you socialize with your neighbors?” “How often do you get together with other friends?” The rating in terms of frequency on a 5-point scale ranged from “never” (=1), “a few times a year or less” (=2), “several times a month” (=3), “several times a week” (=4), and “every day” (=5).

### Control variables

We controlled for demographic variables such as gender, age, education level, physical health status and place of residence of the respondents. Of these, gender [“male” (=0), “female” (=1)], and educational attainment were measured linearly using the respondent’s total years of education; physical health status was an ordinal variable [“very unhealthy” (=1), “relatively unhealthy” (=2), “fair” (=3), “relatively healthy” (=4), and “very healthy” (=5)]; place of residence [“rural” (=0), “urban”(=1)].

### Statistical analysis

Descriptive statistics and Pearson correlation analyses were performed using STATA 17.0 software (two-sided test *p* < 0.05 for significant correlation); we then performed OLS regressions controlling for gender, education, age, place of residence, and socioeconomic status in order to test for a relationship between Internet use, social support, and physical activity. All control variables were hypothesized to have an effect on participation in physical activity. Finally, multiple mediated effects were analyzed in SPSS 27.0 using Model 4 in the process plug-in developed by Hayes (67), and confidence intervals for each effect were established by estimating direct and indirect effects through bootstrap testing with repeated sampling of the sample data. When the confidence interval is not zero, the corresponding effect is significant.

## Results

### Descriptive statistics

Descriptive statistics of the key variables are given in Table 1. The age of the respondents ranged from 60 to 99 years with a mean age of 70.2 years. Among them, 51.3% were old adults females and 48.7% were males. There were more respondents living in rural areas than in urban areas, with 59.93% rural residents, 40.7% urban. old adults people were in good health and generally had low levels of education, with 20% having high school and above. The average sports participation score was 2.729 (SD = 1.787). Notably, more than half of the older adults surveyed had never used the Internet (61.3%). The mean score for relative support was 1.915 (SD = 0.778), friend support was 1.874 (SD = 1.097), and neighborhood support was 2.926 (SD = 1.504).

**Table 1:** Basic variable description statistics table.

Variable	Obs	Mean	Std. Dev.	Min	Max
Physical exercise	2,885	2.729	1.787	1	5
Internet use	2,885	2.085	1.533	1	5
Relative support	2,885	1.915	0.778	1	5
Friend support	2,885	1.974	1.097	1	5
Neighbors support	2,885	2.926	1.504	1	5
Gender	2,885	0.513	0.5	0	1
Age	2,885	70.194	6.844	60	99
Urban	2,885	0.401	0.49	0	1
Health	2,885	3.073	1.124	1	5
Education	2,885	8.183	2.804	6	19

### Relationship between internet use and physical activity

The results of Table 2, which shows our main model, show that all nested regression models were statistically significant between models. Model 1 serves as the baseline model and only tests the effect of control variables on physical activity. We can clearly see that age, place of residence, physical fitness and education have a significant effect on the participation of older adults in physical activity, but there is no gender difference in physical activity among older adults.

**Table 2:** Impact of internet use on physical activity among Chinese older adults: based on OLS model.

Variable	Model 1	Model 2	Model 3	Model 4	Model 5
Gender	−0.054 (0.064)	−0.067 (0.064)	−0.067 (0.064)	−0.067 (0.064)	−0.077 (0.064)
Age	−0.015*** (0.005)	−0.011** (0.005)	−0.011** (0.005)	−0.011** (0.005)	−0.012** (0.005)
Urban	0.671*** (0.073)	0.620*** (0.074)	0.595*** (0.074)	0.586*** (0.074)	0.600*** (0.074)
Health	0.182*** (0.028)	0.171*** (0.029)	0.160*** (0.029)	0.154*** (0.029)	0.152*** (0.029)
Education	0.098*** (0.013)	0.081*** (0.014)	0.079*** (0.014)	0.080*** (0.014)	0.081*** (0.014)
Internet use		0.099*** (0.024)	0.093*** (0.024)	0.088*** (0.024)	0.089*** (0.024)
Relative support			0.153*** (0.041)	0.105** (0.044)	0.098** (0.044)
Friend support				0.089** (0.031)	0.073** (0.032)
Neighbors support					0.043** (0.022)
_cons	2.196*** (0.384)	1.879*** (0.390)	1.672*** (0.393)	1.636*** (0.393)	1.564*** (0.394)
*N*	2,885	2,885	2,885	2,885	2,885
*pseudo R^2^*	0.114	0.120	0.124	0.126	0.127
BIC	11235.226	11225.754	11219.866	11219.653	11223.753

Standard errors in parentheses. *p < 0.1, **p < 0.05, ***p < 0.001.

Model 2 examines the impact of internet use on physical activity. The results indicate that the regression coefficient of internet use on physical activity is 0.099, which is significant at the 0.001 level. This finding suggests a strong correlation between the two variables. In other words, the more frequently older adults use the internet, the more often they engage in physical activity. Therefore, Hypothesis 1 (H1) is validated.

Models 3, 4, and 5 analyze the effects of three dimensions of social support—kin support, friend support, and neighbor support—on old adults individuals’ participation in physical activity. The results indicate that kin support (β = 0.153, *p* < 0.001), friend support (β = 0.089, *p* < 0.05), and neighbor support (β = 0.043, *p* < 0.05) are all positively correlated with physical activity among the old adults. Among these, kin support has the most substantial influence on old adults individuals’ participation in physical activity, followed by friend support, and finally, neighbor support. Consequently, H2 is confirmed. Additionally, the regression analysis reveals that among the control variables, excluding gender, age, residence, physical health, and educational attainment all pass significance tests across the five models. This finding suggests that physical activity among the old adults is not significantly related to gender, but is significantly associated with the other control variables. Overall, younger old adults individuals, those living in urban areas, those in good health, and those with higher educational attainment are more likely to engage in physical activity more frequently.

### Mediation effect analysis

The results presented in the first and second sections of Table 3 indicate that the coefficients for internet use’s impact on social support are 1.356, 1.140, and 2.281, respectively, with 95% confidence intervals (CI) not containing zero. Similarly, the coefficients for social support’s impact on physical activity are 0.098, 0.073, and 0.043, with corresponding 95% (CI) also not containing zero. These findings suggest that social support serves as a partial mediator in the relationship between internet use and participation in physical activity. To further examine the multiple mediation effects of social support, PROCESS Model 4 was employed. According to the total effects outlined in Table 4, internet use has a significant positive impact on physical activity [Bootstrap 95% CI: 0.0525, 0.1453]. The direct effects analysis also reveals a significant positive relationship between internet use and physical activity [Bootstrap 95% CI: 0.0421, 0.1350]. Regarding the influence of social support on physical activity, the indirect effects show that two types of social support—kin support and friend support—have significant positive effects on physical activity among old adults individuals in China ([Bootstrap 95% CI: 0.0002, 0.0090]; [Bootstrap 95% CI: 0.0003, 0.0114]). However, neighbor support does not have a significant effect [Bootstrap 95% CI: −0.0008, 0.0037], partially supporting H3. Overall, two significant mechanisms emerge through the indirect pathway of social support: (1) internet use → kin support → physical activity and (2) internet use → friend support → physical activity. Therefore, we conclude that kin support and friend support mediate the relationship between internet use and physical activity.

**Table 3:** Path-coefficients of the mediating models.

Variables	β	BC95%LL	BC95%UL	R^2^	*F*
Internet use vs. Social support					
Internet use → Relative support	1.356	1.006	1.703	0.045	22.808
Internet use → Friend support	1.140	0.649	1.631	0.039	19.203
Internet use → Neighbors support	2.281	1.603	2.959	0.023	11.441
Social support vs. Physical exercise					
Relative support → Physical exercise	0.098	0.011	0.186		
Friend support → Physical exercise	0.073	0.010	0.137		
Neighbors support → Physical exercise	0.043	0.0001	0.087		
Internet use vs. Physical exercise					
Internet use → Physical exercise	0.089	0.042	0.135		

**Table 4:** Mediating effects of social support.

Paths	Standardized coef.	Bootstrap 95%CI	
		Lower	Upper
Total effect			
Internet use → Physical exercise	0.0989	0.0525	0.1453
Direct effects			
Internet use → Physical exercise	0.0886	0.0421	0.1350
Indirect effects (total)	0.0103	0.0045	0.0172
Internet use → Relative support → Physical exercise	0.0041	0.0002	0.0090
Internet use → Friend support → Physical exercise	0.0052	0.0003	0.0114
Internet use → Neighbors support → Physical exercise	0.0010	−0.0008	0.0037

## Discussion

In the context of a comprehensive response to the challenges of population aging in the new era, this study utilizes data from the China General Social Survey (CGSS2021) to examine the impact of internet use on physical activity participation among the old adults in China. While previous research has explored the relationship between internet use and physical activity, the underlying mechanisms of this process remain insufficiently understood. To fill this gap, we propose a multiple mediator model to investigate the role of social support. The results of this study show that Internet use is positively associated with physical activity by coming to promote social support. Several important conclusions can be drawn from this study.

First, our findings reveal a positive correlation between internet use and physical activity participation among the old adults, consistent with previous studies (42). Specifically, the internet provides a wealth of information related to sports and health, offering older adults more convenient access to resources such as sports policy promotion, cultural dissemination, and services. This access significantly influences their perceptions and attitudes toward physical exercise, thereby enhancing their willingness to engage in physical activities (11). Additionally, internet use expands the social networks of older adults, facilitating exchanges and interactions with family and friends, through which they receive encouragement and advice regarding exercise, thereby effectively increasing their physical activity. Previous research has also highlighted that activities like “reading news,” “chatting online,” and “watching videos” can enhance public access to sports information and knowledge, subsequently influencing exercise behaviors (41). Our findings further demonstrate the positive role of internet use in promoting physical activity among the old adults, and also validate the effectiveness of China’s “Internet + Exercise” initiative. Therefore, Chinese government agencies can enhance older adults’ fitness awareness and promote their physical activity behaviors by increasing internet accessibility and disseminating more scientific exercise and health information.

Secondly, this study reveals that all three dimensions of social support—namely, support from relatives, friends, and neighbors—have a significant positive impact on old adults individuals’ participation in physical exercise. This finding represents a novel contribution to the existing literature. Previous research has emphasized that social support from relatives and friends plays a dominant role in influencing old adults individuals’ engagement in physical activities (62), and has identified these forms of support as key drivers of their participation in exercise (63). However, our findings further illuminate the critical role of neighbor support in encouraging old adults participation in physical activities. We contend that social support, as an external form of assistance, can positively influence physical activity among the old adults, thereby enhancing their exercise behaviors.

Finally, this study examined the mediating effects of the three dimensions of social support—support from relatives, friends, and neighbors—on the relationship between internet use and old adults individuals’ physical activity. The results indicate that while all three dimensions of social support significantly influence the relationship between internet use and physical activity among the old adults, only support from relatives and friends emerged as significant mediators in this relationship. In contrast, neighbor support did not exhibit a significant mediating effect. His result may be due to the fact that, On one hand, with the empowering and enabling effect of the Internet, the frequency of the old adults using the Internet has increased significantly, which not only effectively enhances their communication and interaction with family and friends (64) but also provides them with more opportunities to share exercise-related information and fitness videos with their relatives and friends, thereby positively influencing their participation in physical activities. On the other hand, the internet offers a variety of exercise modalities for the old adults, such as downloading fitness apps, allowing them to engage in flexible, science-based exercise routines tailored to their individual fitness needs and leisure time (65, 66), thereby reducing their reliance on traditional neighbor support. However, it is important to note that while the internet provides the old adults with abundant fitness resources and platforms, face-to-face interactions, shared physical activities, and mutual encouragement and support from neighbors remain vital. Therefore, in promoting physical activity among the old adults, it is essential to leverage the advantages of the internet while also recognizing the importance of neighbor support.

This study has several limitations that could be addressed in future research. Firstly, due to the constraints of the existing data, this study measures older adults’ internet usage solely from the perspective of frequency, without fully capturing the diversity of internet use, such as duration, psychological motivations, and browsing activities. Secondly, from the perspective of social support, in addition to kin support, friend support, and neighbor support, family support is also a significant influencing factor. Specifically, financial support provided by family members can play a positive role in promoting internet use and physical exercise among older adults. Future research could incorporate family support as a core dimension for more in-depth analysis. Thirdly, causal inferences cannot be solely based on path analysis. Future research should incorporate longitudinal designs or experimental research approaches and could benefit from qualitative methods, such as interviews, to further explore the underlying narratives of the study’s findings and enhance the richness of the results.

## Conclusion

This study, based on the CGSS 2021 survey data, integrates internet usage, social support, and physical exercise into a unified framework and specifically examines the mediating role of various dimensions of social support in the relationship between internet usage and physical exercise among older adults. The findings reveal that: first, internet usage can enhance older adults’ participation in physical exercise; second, all three dimensions of social support—namely, kin support, friend support, and neighbor support—positively influence older adults’ engagement in physical exercise; and third, two aspects of social support, relatives and friends, played a partly mediating role in the process. Through the internet, older adults in China can maintain contact with geographically dispersed relatives and friends, thereby increasing communication with kin and friends, which facilitates their active participation in physical exercise. In conclusion, given the positive impacts of both internet usage and social support on older adults’ engagement in physical exercise, it is imperative for the government to implement effective measures to encourage and enhance internet usage among older adults and to strengthen various forms of social support. This approach will promote increased physical activity among the old adults and contribute to addressing the challenges posed by population aging.

## Data Availability

The original contributions presented in the study are included in the article/supplementary material, further inquiries can be directed to the corresponding author.
